# Multicenter Study of Multimodal MRI Radiomics and Deep Learning-Based Segmentation for Predicting Local Recurrence of Nasopharyngeal Carcinoma

**DOI:** 10.3390/cancers18081265

**Published:** 2026-04-16

**Authors:** Dongfang Yao, Yongjing Lai, Xiang Bin, Jingyu Li, Biaoyou Chen, Anzhou Tang

**Affiliations:** 1 Department of Otolaryngology–Head and Neck Surgery, The First Affiliated Hospital of Guangxi Medical University, Nanning 530021, China; 2 The Affiliated Tumor Hospital of Guangxi Medical University, Nanning 530021, China; 3 Key Laboratory of Early Prevention and Treatment for Regional High Frequency Tumor, Guangxi Medical University, Ministry of Education, Nanning 530021, China

**Keywords:** nasopharyngeal carcinoma, magnetic resonance imaging, radiomics, deep learning, automatic segmentation, Swin UNet, recurrence prediction

## Abstract

**Background/Objectives**: We developed and validated a multimodal magnetic resonance imaging (MRI) framework combining deep learning segmentation with radiomics to predict local recurrence in nasopharyngeal carcinoma (NPC). **Methods**: This retrospective two-center study included 1074 NPC patients treated between 2015 and 2019. Center 1 cases were split 8:2 into training and internal test sets, while Center 2 served for external validation. A multimodal Swin UNet model automatically segmented tumors from pretreatment T1-weighted, T2-weighted, and contrast-enhanced T1 (CET1) images. Radiomics features were extracted from expert-reviewed regions of interest, selected, and modeled using extreme gradient boosting for recurrence prediction. **Results**: The multimodal segmentation model maintained consistent but moderate Dice similarity coefficients (0.737, 0.666, and 0.726 for T1WI, T2WI, and CET1 in external validation). These values reflect the moderate overlap typical for nasopharyngeal carcinoma, given its highly infiltrative growth and ill-defined boundaries along complex anatomic interfaces. For local recurrence prediction, single-modality models reached external AUCs between 0.754 and 0.781. Importantly, the multimodal fusion model demonstrated numerical improvement over single modalities in the external validation set (e.g., vs. T1WI, *p* = 0.141), achieving an AUC of 0.910, accuracy of 0.908, sensitivity of 0.805, specificity of 0.946, and F1-score of 0.825. **Conclusions**: The multimodal MRI radiomics model, developed alongside a deep learning segmentation module, demonstrated favorable multicenter performance for evaluating NPC recurrence risk. The primary prognostic analysis was based on expert-reviewed regions of interest; a supplementary analysis using fully automatic segmentation masks yielded comparable, non-significantly different performance across all cohorts (Training AUC: 0.887; Internal Test AUC: 0.892; External Validation AUC: 0.885 vs. 0.910, *p* = 0.145), supporting the feasibility of future end-to-end deployment. Fusing multimodal features yielded numerical improvements over single-sequence models in external validation, providing a basis for post-treatment surveillance planning.

## Simple Summary

Predicting local recurrence in nasopharyngeal carcinoma (NPC) remains challenging using standard imaging assessment alone. This study developed and externally validated a prognostic framework using multimodal MRI radiomics based on expert-reviewed tumor regions and supplemented this by evaluating an automated deep learning tumor segmentation pipeline to enhance recurrence risk evaluation. Analyzing data from 1074 patients across two clinical centers, we found that integrating T1-weighted, T2-weighted, and contrast-enhanced MRI signals captures complementary features of intratumoral heterogeneity. While the deep learning segmentation module demonstrated consistent but moderate overlap with expert contours (reflecting the complex infiltrative boundaries of NPC), the derived prognostic model achieved high discriminative performance in an independent external validation cohort (AUC: 0.910). The multimodal model showed numerical improvement over single-sequence approaches, suggesting that fusing diverse imaging signals provides a more comprehensive assessment of individual recurrence risk. This objective tool may support personalized surveillance planning and prospective clinical decision-making for NPC patients.

## 1. Introduction

Nasopharyngeal carcinoma (NPC) is a regionally prevalent head and neck malignancy. According to the latest GLOBOCAN estimates, NPC accounts for approximately 73,400 deaths annually worldwide, with the vast majority of this mortality burden concentrated in endemic regions of Asia [1]. Although intensity-modulated radiotherapy (IMRT) and combined systemic treatment have improved local control, local recurrence remains a clinically important cause of treatment failure [2,3,4]. Accurate identification of patients at high risk of recurrence is therefore relevant to surveillance planning and timely salvage treatment [5,6,7].

Magnetic resonance imaging is central to NPC evaluation because of its high soft-tissue contrast and its ability to depict primary tumor extent and adjacent tissue involvement [2,8]. T1-weighted imaging provides anatomic detail, T2-weighted imaging reflects tissue water content and edema, and contrast-enhanced T1-weighted imaging offers information related to enhancement and perfusion. These sequences capture complementary aspects of tumor biology, making multimodal analysis attractive for prognostic modeling [6,9,10]. Recently, multimodal MRI radiomics has been increasingly recognized for its capability to refine personalized risk stratification and prognosis beyond standard clinical staging [11].

Radiomics provides quantitative descriptors of tumor intensity, shape, and texture that may reveal intratumoral heterogeneity beyond visual inspection [12]. However, radiomics pipelines depend heavily on the quality and consistency of tumor segmentation [13,14,15,16]. In NPC, manual contouring is labor-intensive and subject to observer variability, especially in the setting of irregular anatomy and skull base extension [17,18]. Deep learning segmentation can reduce this burden and improve reproducibility, particularly when multimodal MRI is used as input [18,19,20]. State-of-the-art deep learning architectures have recently demonstrated exceptional precision in automated head and neck tumor delineation, supporting the feasibility of integrating automated segmentation into future imaging pipelines [18].

Several imaging-based prediction studies have been reported in NPC, but robust multicenter evidence for local recurrence prediction remains limited [5,6,21]. In addition, external generalizability remains a central challenge in radiomics research because scanners, acquisition protocols, and patient populations vary across centers [13,15,22]. Recent evidence syntheses also show that MRI- and CT-based radiomics applications in NPC are expanding rapidly, particularly for treatment-response prediction [23]. We therefore developed a two-center workflow that combined multimodal Swin UNet-based segmentation with MRI radiomics and evaluated whether multimodal fusion could improve the prediction of local recurrence compared with single-modality models. In the present study, the segmentation module was developed and evaluated as a dedicated component, whereas radiomics modeling was performed on expert-reviewed tumor regions of interest rather than directly on automatic segmentation masks.

## 2. Materials and Methods

### 2.1. Study Design and Ethics

This retrospective cohort study included two institutions. Patients were enrolled from January 2015 through January 2019, and follow-up was completed through January 2024 to ensure sufficiently mature follow-up for recurrence assessment in the latest enrolled cases, unless local recurrence was confirmed earlier. This specific enrollment timeframe (January 2015 to January 2019) was deliberately selected to ensure more mature follow-up assessment and reduce treatment-era heterogeneity across modern IMRT protocols. The study was approved by the institutional review boards of the participating centers, and the requirement for informed consent was waived because of the retrospective design.

### 2.2. Eligibility Criteria and Endpoint Definition

Patients were eligible if they met the following criteria: (1) histopathologically confirmed nasopharyngeal carcinoma; (2) pretreatment MRI performed within 2 weeks before treatment initiation, including T1WI, T2WI, and CET1; (3) no antitumor therapy before baseline imaging; (4) completion of definitive intensity-modulated radiotherapy with concurrent chemotherapy according to institutional practice; and (5) complete clinical and imaging follow-up for at least 5 years after treatment initiation or until pathologically confirmed local recurrence.

Patients were excluded for distant metastasis at diagnosis, coexistence of another primary malignancy, missing or poor-quality MRI sequences, incomplete follow-up, or failure to complete the planned treatment course.

The primary endpoint was local recurrence at or adjacent to the primary nasopharyngeal site. Suspected recurrence required pathological confirmation by biopsy. Follow-up time was defined from treatment initiation to pathologically confirmed local recurrence or last available follow-up. Follow-up was performed every 3 to 4 months during years 1 and 2, every 6 months during years 3 to 5, and annually thereafter.

### 2.3. Cohort Allocation

**Figure 1:**
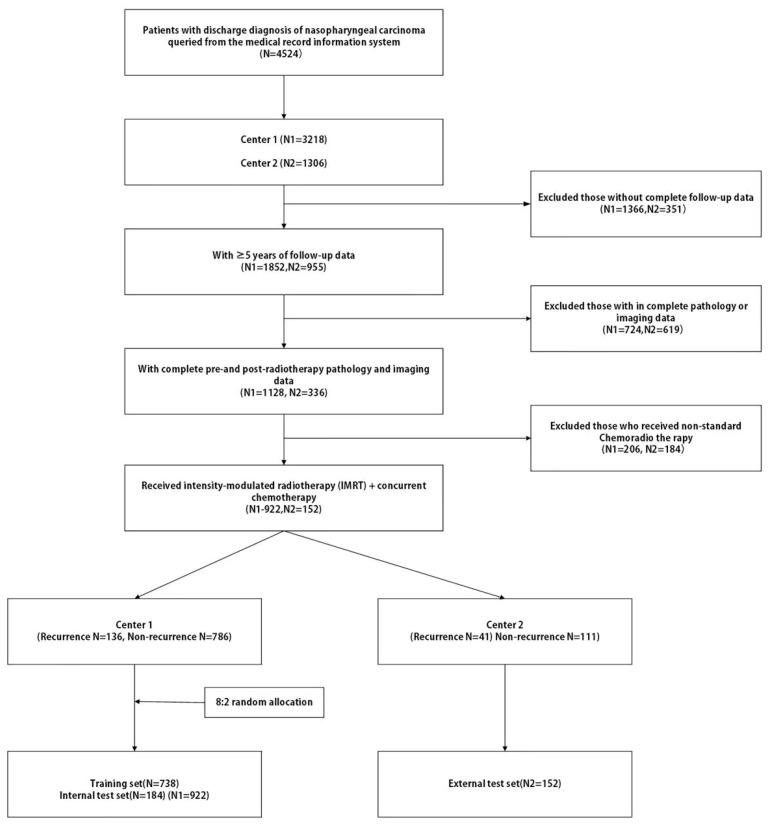
Flowchart of case inclusion, exclusion, and cohort allocation.

Center 1 was used for model development and internal validation. Cases from Center 1 were divided into training and internal test sets using a patient-level stratified random 8:2 split to preserve the recurrence ratio and avoid information leakage. The internal test set was fixed prior to any analysis and remained completely withheld throughout all stages of feature selection, model training, and hyperparameter tuning; it was used solely for post hoc performance evaluation. All cases from Center 2 were reserved for external validation (Figure 1).

### 2.4. MRI Acquisition

A broadly standardized MRI acquisition protocol was used across centers. Patients were examined in the supine position with a head–neck coil. The scanning range extended from the choanae to the clavicles, with additional coronal or sagittal images acquired when needed. For T1-weighted imaging, the repetition time was 1670–2276 ms, the echo time was 6.06–11.12 ms, the flip angle was 90 degrees, the slice thickness was 6 mm, and the slice gap was 1 mm. For T2-weighted imaging, the repetition time was 2620–7420 ms, the echo time was 102–107 ms, the flip angle was 90 degrees, the slice thickness was 6 mm, and the slice gap was 1 mm. For contrast-enhanced T1-weighted imaging, the repetition time was 1866–2612 ms, the echo time was 8.14–11.18 ms, and gadolinium was administered intravenously at 0.1 mmol/kg before acquisition. Both 1.5-T and 3.0-T systems were used in clinical practice at the participating centers, including Ingenia Elition X (Philips Healthcare, Amsterdam, The Netherlands) and MAGNETOM Altea (Siemens Healthineers, Erlangen, Germany).

### 2.5. Image Preprocessing

DICOM data were anonymized and converted to NIfTI format. N4 bias-field correction and isotropic resampling to 1.0 × 1.0 × 1.0 mm were applied. CET1 was used as the registration reference, and T1WI and T2WI were aligned to CET1 using rigid-affine registration driven by mutual information. Image intensities were z-score-normalized, and all volumes were cropped to the nasopharyngeal region before model training and feature extraction. All image preprocessing procedures described above were implemented in Python using SimpleITK (version 2.5.2).

### 2.6. Reference Segmentation and Reproducibility Assessment

Primary tumors were manually delineated in ITK-SNAP (version 3.8.0) by a head and neck surgeon with more than 10 years of experience. Two senior radiologists reviewed the contours and resolved disagreements by consensus. For radiomics reproducibility analysis, a random subset of 50 patient cases from Center 1 was selected for repeat annotation. To evaluate intra-observer reproducibility, Radiologist A (10 years of experience) performed a second round of segmentation with a 2-week interval between measurements. To evaluate inter-observer reproducibility, Radiologist B (8 years of experience) independently delineated the same 50 cases. Intraclass correlation coefficients (ICC) were calculated using a two-way mixed-effects model for absolute agreement (ICC(2,1)). Only features achieving both intra-observer and inter-observer ICC values greater than 0.80 were considered stable and retained for subsequent modeling.

### 2.7. Automatic Segmentation Model

**Figure 2:**
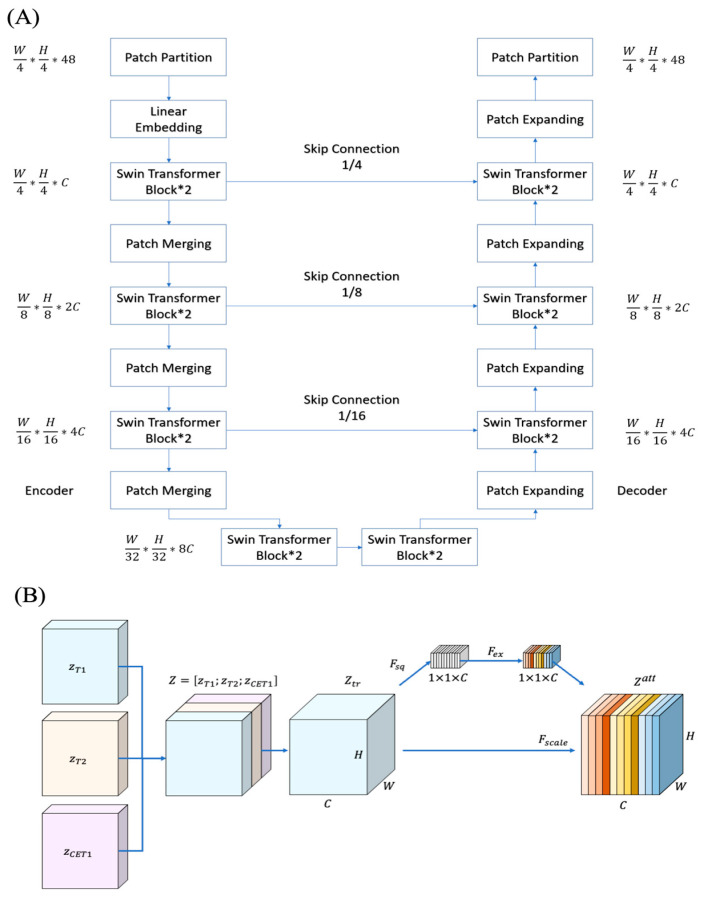
Multimodal Swin UNet architecture and fusion strategy. (**A**) Architecture of the Swin Transformer-based encoder–decoder framework. (**B**) Diagram of the multimodal feature fusion and attention weighting module.

A multimodal Swin UNet architecture was used for automatic tumor segmentation (Figure 2). The model was trained in a 2.5-dimensional manner, using axial slices with adjacent slices stacked as input channels (e.g., a 9-channel input for 3 adjacent slices across 3 MRI modalities). T1WI, T2WI, and CET1 were combined as multimodal inputs. The network used a Swin Transformer encoder and a U-Net-style decoder with skip connections to preserve spatial detail.

Input images were resized to 256 × 256. Early multimodal fusion was performed by channel concatenation followed by convolutional projection. The loss function combined Dice loss, cross-entropy loss, and a boundary-aware term. Training used the AdamW optimizer with an initial learning rate of 1 × 10^−4^, weight decay of 1 × 10^−4^, cosine annealing, early stopping, and standard image augmentation including flipping, rotation, scaling, translation, elastic deformation, Gaussian noise, and brightness or contrast perturbation.

Segmentation performance was evaluated using the Dice similarity coefficient. Representative gradient-weighted class activation mapping (Grad-CAM) visualizations were used to illustrate the image regions contributing to model attention.

### 2.8. Radiomics Feature Extraction and Selection

Radiomic features were extracted from expert-reviewed tumor regions of interest using PyRadiomics (version 3.0.1) [24]. In this study, the radiomics analysis was based on these reference contours rather than directly on automatic segmentation masks. Feature classes included first-order, shape, gray-level co-occurrence matrix, gray-level run-length matrix, gray-level size-zone matrix, gray-level dependence matrix, and neighboring gray-tone difference matrix features derived from original, Laplacian-of-Gaussian-filtered, and wavelet-transformed images.

To improve robustness, features with poor reproducibility were removed first. Variance thresholding and Pearson correlation filtering were then applied to remove low-information and redundant features. Finally, the least absolute shrinkage and selection operator (LASSO) with 10-fold cross-validation was used to retain a sparse feature subset for each modality and for the multimodal fusion model. Specifically, a Pearson correlation coefficient cutoff threshold of |r| > 0.90 was utilized. To ensure optimal selection and rigorous evaluation of model generalizability, LASSO was comparatively evaluated against Elastic Net and Ridge Regression, with hyper-parameters identically grid-searched across a strict 10-fold cross-validation framework. All comparative evaluations were restrained to identical patient subset partitions (Training, Internal, and External Test sets) to yield an objective comparison. All feature selection procedures, including ICC-based reproducibility filtering, variance thresholding, Pearson correlation filtering, and LASSO regularization with 10-fold cross-validation, were performed exclusively on the training set. No information from the internal test set or the external validation set was used at any step of feature selection or hyperparameter optimization.

### 2.9. Prognostic Modeling

Extreme gradient boosting was used to construct recurrence prediction models for T1WI, T2WI, CET1, and fused multimodal radiomics. Hyperparameters were optimized through cross-validation in the training cohort. Class imbalance was handled using class weighting, and the final decision threshold was determined in the development cohort and then applied unchanged to both test cohorts.

Model performance was summarized using AUC, accuracy, sensitivity, specificity, positive predictive value, negative predictive value, and F1-score. AUCs were compared using the DeLong test, and classification agreement between models was assessed using McNemar’s test.

### 2.10. Statistical Analysis

All analyses and model development were performed using Python (version 3.9.12), with PyTorch (version 1.11.0) and scikit-learn (version 1.0.2). The custom scripts and codes underlying this article are available from the corresponding author upon reasonable request. Continuous variables were compared using independent-samples *t*-tests or Mann–Whitney U tests, as appropriate. Categorical variables were compared using chi-square tests or Fisher exact tests. Ordinal variables were analyzed using nonparametric methods when distributional assumptions for parametric testing were not satisfied. A two-sided *p* value below 0.05 was considered statistically significant.

## 3. Results

### 3.1. Patient Selection and Baseline Characteristics

Among 4524 screened cases, 1074 met the inclusion criteria. Center 1 contributed 922 patients, including 136 recurrence events and 786 nonrecurrence cases, and was used for model development and internal testing. Center 2 contributed 152 patients, including 41 recurrence events and 111 nonrecurrence cases, and was used for external validation (Figure 1).

The baseline characteristics of the recurrence and nonrecurrence groups were broadly comparable in age, sex distribution, radiotherapy parameters, and chemotherapy exposure. Most patients had stage III to IVa disease. Detailed demographic and clinical data are summarized in Table 1.

### 3.2. Automatic Segmentation Performance

**Figure 3:**
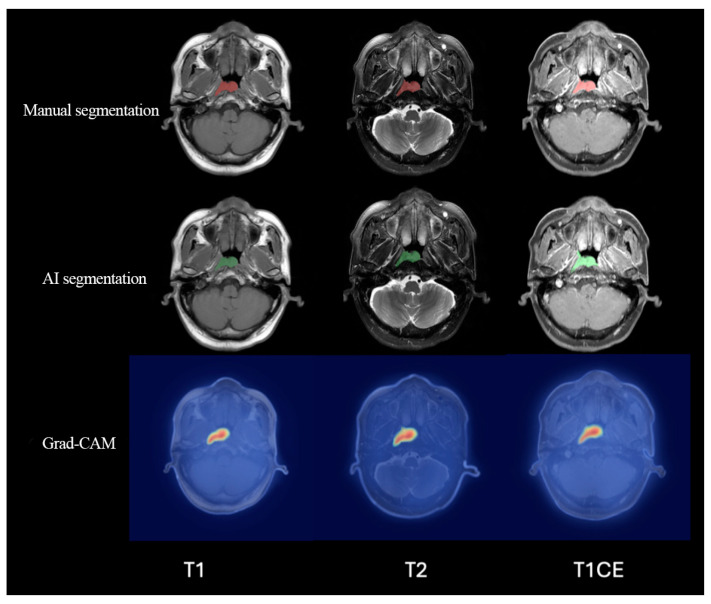
Representative comparisons between expert contours and automatic segmentation, with Grad-CAM visualization. Abbreviations: Grad-CAM, gradient-weighted class activation mapping.

The Swin UNet model achieved Dice similarity coefficients of 0.709, 0.679, and 0.654 for T1WI, T2WI, and CET1 in the training set. The corresponding Dice scores in the internal test set were 0.644, 0.647, and 0.701, and those in the external test set were 0.737, 0.666, and 0.726. Visual comparison with expert contours showed generally close overlap, and Grad-CAM maps highlighted clinically relevant tumor regions (Table 2, Figure 3).

### 3.3. Radiomics Feature Selection

**Figure 4:**
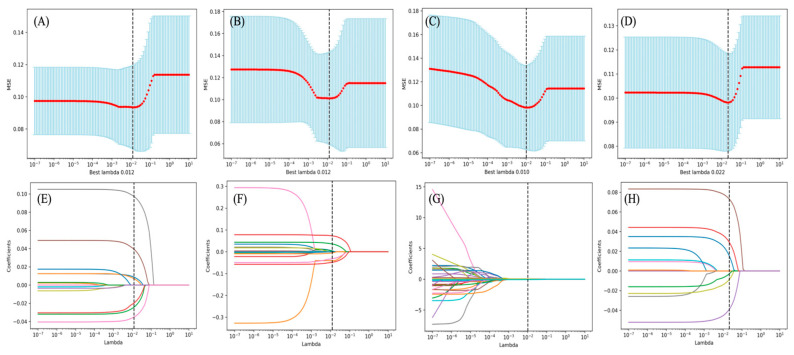
LASSO-based radiomics feature selection for T1WI, T2WI, CET1, and multimodal fusion. Abbreviations: LASSO, least absolute shrinkage and selection operator. (**A**) T1WI: cross-validation curve. (**B**) T2WI: cross-validation curve. (**C**) CET1: cross-validation curve. (**D**) Multimodal fusion: cross-validation curve. (**E**) T1WI: coefficient profile. (**F**) T2WI: coefficient profile. (**G**) CET1: coefficient profile. (**H**) Multimodal fusion: coefficient profile.

For each modality, 1316 radiomic features were initially extracted. After reproducibility filtering, redundancy reduction, and LASSO-based selection, 7 features were retained for T1WI, 10 for T2WI, and 10 for CET1. The multimodal fusion model retained 6 core features spanning shape, first-order, and texture domains (Table 3, Figure 4). In the filtering cascade, after ICC filtering and subsequent variance thresholding, 1206, 1189, and 1215 features remained for T1WI, T2WI, and CET1, respectively. The Pearson correlation filtering removed 1064, 1033, and 1067 highly correlated features, effectively leaving 142, 156, and 148 features (Table S1). Bypassing this Pearson filtering step led to overfitting on the training set (AUC 0.932 vs. 0.905) and severely degraded validation performance, with AUCs dropping to 0.885 in the internal test set and 0.864 in the external validation cohort (*p* = 0.035, DeLong test) (Figure S9). Furthermore, when evaluating shrinkage algorithms, Elastic Net selected 15 features yielding an external AUC of 0.895, while Ridge regression, which retained all pooled features, showed inferior external validation performance (0.831 AUC) (Figure S11). Thus, LASSO effectively eliminated redundant variables and demonstrated better generalizability. Quantitative independence of the six finally selected multimodal variables was corroborated by Variance Inflation Factors strictly below 5 (ranging from 1.45 to 3.64) (Table S2). Detailed methodology, parameters grids, data partitions, and complete numerical subsets are comprehensively documented in the Supplementary Materials.

### 3.4. Single-Modality Prediction Performance

**Figure 5:**
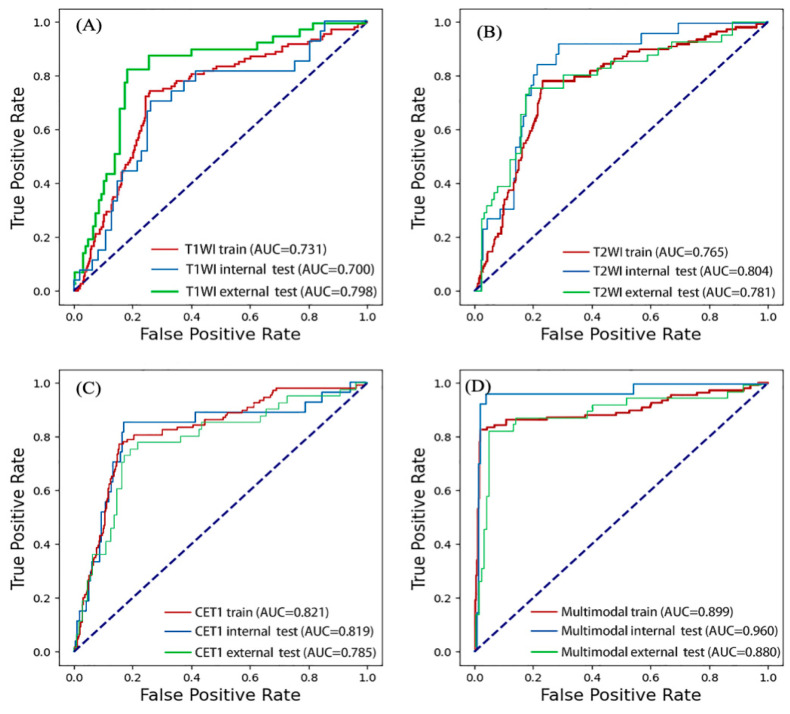
Receiver operating characteristic curves for the single-modality and multimodal models. (**A**) T1WI: ROC curves in the training, internal test, and external test sets. (**B**) T2WI: ROC curves in the training, internal test, and external test sets. (**C**) CET1: ROC curves in the training, internal test, and external test sets. (**D**) Multimodal fusion: ROC curves in the training, internal test, and external test sets.

For T1WI, the AUCs were 0.773 in the training set, 0.776 in the internal test set, and 0.781 in the external test set (Figures S1 and S5). For T2WI, the corresponding AUCs were 0.752, 0.660, and 0.754 (Figures S2 and S6). For CET1, the AUCs were 0.853, 0.867, and 0.775 (Figures S3 and S7). On the external test set, the T1WI and T2WI models achieved accuracies of 0.776 and 0.763, with F1-scores of 0.667 and 0.640, respectively. CET1 showed relatively strong performance in development but a larger drop in external validation (Table 4, Figure 5).

### 3.5. Multimodal Fusion Model Performance

The multimodal fusion model achieved AUCs of 0.905 in the training set, 0.913 in the internal test set, and 0.910 in the external test set (Figure S4). In the external test set, accuracy was 0.908, sensitivity was 0.805, specificity was 0.946, positive predictive value was 0.846, negative predictive value was 0.929, and F1-score was 0.825. Receiver operating characteristic curves and confusion matrices indicated improved discrimination compared with the single-modality models, although the AUC improvements in the external set were numerical and did not reach statistical significance in all comparisons (Table 4, Figure 5 and Figure S8).

### 3.6. Comparative Effectiveness

Compared with the single-modality models, the multimodal fusion model showed significantly higher AUCs in the training and internal test cohorts, particularly relative to T1WI and T2WI. In the external cohort, the numerical advantage of the fusion model remained evident, although some pairwise AUC differences did not reach statistical significance. McNemar testing nevertheless suggested better classification consistency for the fusion model than for T1WI and T2WI (Table 5).

## 4. Discussion

This study developed and externally tested a two-center workflow for predicting local recurrence of nasopharyngeal carcinoma using multimodal MRI radiomics and deep learning-based segmentation. The main finding was that multimodal fusion showed numerical improvement over the single-modality models and maintained favorable performance in an independent external cohort. This result extends prior NPC radiomics studies addressing local recurrence, recurrence-related risk, and MRI-based prognostic modeling in smaller or less externally validated cohorts [5,6,21]. Additional recent work has addressed CT-based local recurrence prediction [25], early response assessment after concurrent chemoradiotherapy [26], and recurrence prediction after neoadjuvant treatment in multicenter settings [27].

The segmentation component showed consistent but stable Dice performance across centers. This is relevant because NPC often extends along complex anatomic interfaces, which can make manual delineation difficult and reduce consistency across readers [17,18,20]. The use of a multimodal Swin UNet architecture likely contributed to robust boundary recognition by combining complementary sequence information with long-range contextual modeling [19,20,28]. Grad-CAM visualization was included as an interpretability aid for the segmentation module [29]. Although the primary prognostic analysis was based on expert-reviewed regions of interest, our supplementary integration analysis demonstrated that substituting these expert contours with fully automatic segmentation masks resulted in extremely slight, non-significant attenuation in prognostic discrimination (AUCs of 0.887, 0.892, and 0.885 in the training, internal test, and external validation cohorts, respectively, compared to 0.905, 0.913, and 0.910 based on expert-reviewed contours; *p* = 0.145) (Figure S13). This supports the feasibility of future end-to-end clinical deployment without significant loss of prognostic value.

The prognostic results support the value of multimodal integration. T1WI, T2WI, and CET1 each captured distinct aspects of tumor phenotype, but no single sequence fully represented recurrence-related heterogeneity. The fusion model combined these complementary signals and achieved the best discrimination, with especially strong specificity in the external cohort. This pattern is consistent with the prior radiomics literature showing the potential value of multimodal descriptors over more limited single-sequence analysis [10,30,31]. Other recent studies have likewise emphasized multisequence MRI radiomics for outcome stratification [32,33], as well as integration of radiomics with deep learning for response prediction [34]. Earlier MRI-based work also explored biological risk stratification in NPC [35]. The weaker cross-center transferability of the CET1-only model also suggests that contrast-enhanced features may be more sensitive to acquisition heterogeneity than fused multimodal descriptors [13,15,22]. The stable and slightly higher performance in the external cohort may also be partly related to its higher local recurrence event rate, which may have yielded a clearer separation between recurrent and nonrecurrent cases. Moreover, despite the algorithmic coexistence of mathematically parallel first-order variables strictly retained by LASSO (e.g., Median, Range, and Interquartile Range), rigorous leave-out ablation studies supported their distinct prognostic contributions. Specifically, the targeted ablation of dispersion parameters markedly degraded performance (yielding AUCs of 0.892, 0.890, and 0.882 in the training, internal test, and external validation cohorts, respectively, vs. 0.905, 0.913, and 0.910 for the baseline), suggesting that they encompass complementary prognostic profiles for capturing clinical heterogeneity (Figure S10).

From a methodological perspective, several preprocessing choices were critical to ensuring cross-center robustness. Regarding image preprocessing, the images were resampled to 1.0 × 1.0 × 1.0 mm. While this standardized voxel spacing, it may introduce interpolation artifacts. However, our internal ablation confirmed that bypassing this step significantly deteriorated multi-institutional validation performance (External AUC dropped from 0.910 to 0.841, *p* = 0.012), indicating that isotropic resampling represents a necessary algorithmic tradeoff for maintaining multicenter generalizability (Figure S14). Importantly, the final multimodal signature consisted predominantly of first-order and 2D shape features, which may partly explain its relative robustness to through-plane resampling. Furthermore, although the MRI protocol was harmonized as much as possible, scanner vendors, field strengths, and acquisition details were not completely identical across centers. To ensure robustness, we performed an additional ComBat-based harmonization analysis, which yielded highly consistent feature selection and non-significant shifts in external validation (AUC 0.912 vs. 0.910, *p* = 0.925) (Figure S12). This suggests that scanner-related bias is unlikely to fully explain the observed performance.

From a clinical perspective, a reliable recurrence-risk model could help identify patients who may benefit from closer surveillance after definitive treatment. This is clinically relevant because recurrence after IMRT remains an important source of treatment failure in NPC, and most failures occur within the first few years after treatment [3,7,36]. The present results support the feasibility of combining MRI-based quantitative analysis with deep learning-based segmentation research, although further validation is needed before a fully automated clinical pipeline can be claimed. Compared with recently reported approaches that incorporate both pre- and post-treatment longitudinal MRI for outcome prediction (e.g., Dang et al. [30], which achieved an external validation AUC of 0.827), the present framework relies exclusively on pretreatment imaging. This offers a distinct advantage for pre-treatment risk stratification by supporting clinical assessment prior to treatment initiation, while maintaining a complementary relationship with longitudinal imaging strategies.

Several limitations should be noted. The number of recurrence events in the external cohort was limited, which restricts the precision of subgroup analysis and effect estimation. Additionally, we acknowledge the substantial difference in local recurrence prevalence between Center 1 (14.8%) and Center 2 (27.0%), primarily reflecting the referral nature of Center 2 as a specialized center. While prevalence-dependent metrics may shift, our core discriminative indices—including AUC, sensitivity, and specificity—are mathematically independent of disease prevalence. The consistent AUC in the external cohort suggests that the model retained discriminative ability in a higher-prevalence referral setting, though further validation in broader community-based cohorts with lower baseline prevalence is necessary. Finally, the present model relied only on imaging features and did not incorporate clinical or biological covariates such as Epstein–Barr virus (EBV) DNA, detailed TNM risk structure, or pathological subtype; prior studies suggest that integrating imaging and clinical variables can further improve predictive performance [25,32,33]. While plasma EBV DNA is an important prognostic factor, these measurements were partially missing across our historical multicenter cohorts, and enforcing complete cases would have substantially reduced the analyzable sample size. Recent work also indicates that MRI radiomics and deep feature fusion may be useful in immunochemotherapy and chemoradiotherapy response prediction [34,37], while earlier multiparametric MRI studies also reported prognostic value in advanced NPC [38].

## 5. Conclusions

Multimodal MRI radiomics showed favorable multicenter performance for predicting local recurrence in nasopharyngeal carcinoma. The accompanying deep learning segmentation module demonstrated consistent but moderate performance across centers. The primary prognostic analysis was based on expert-reviewed regions of interest, and the multimodal model demonstrated numerical improvement over single-modality approaches in the external validation set. A supplementary analysis using automatically generated segmentation masks yielded similar prognostic performance, supporting the feasibility of future end-to-end pipeline integration.

## Figures and Tables

**Table 1:** Demographic and clinical characteristics of regularly followed-up patients with nasopharyngeal carcinoma.

Metrics	Center 1 (Training and Internal Test Set)		Center 2 (External Test Set)	
Groups	Recurrence	Non-recurrence	Recurrence	Non-recurrence
Total number of participants	136	786	41	111
Age	47.3 (8~83)	47.2 (8~82)	47.2 (12~77)	47.1 (11~82)
**Sex**				
Male	94 (69.1%)	528 (67.2%)	27 (65.9%)	83 (74.8%)
Female	42 (30.9%)	258 (32.8%)	14 (34.1%)	28 (25.2%)
**Clinical staging**				
I	1 (0.7%)	0 (0.0%)	0 (0.0%)	0 (0.0%)
II	8 (5.9%)	138 (17.6%)	1 (2.4%)	9 (8.1%)
III	57 (42.0%)	301 (38.2%)	19 (46.3%)	46 (41.4%)
IVa	70 (51.4%)	347 (44.1%)	21 (51.2%)	56 (50.5%)
T staging				
T1	(0.0%)	39 (5.0%)	3 (7.3%)	0 (0.0%)
T2	24 (17.6%)	141 (17.9%)	7 (17.1%)	12 (10.8%)
T3	52 (38.2%)	331 (42.1%)	19 (46.3%)	37 (33.3%)
T4	60 (44.1%)	275 (35%)	12 (29.3%)	62 (55.9%)
**Induction or adjuvant chemotherapy**				
Yes	127 (93.4%)	724 (92.2%)	38 (92.7%)	103 (92.8%)
No	9 (6.6%)	62 (7.8%)	3 (7.3%)	8 (7.2%)
Radiation dose (Gy)	70.1 ± 5.5	60.2 ± 6.7	70.3 ± 2.3	70.5 ± 4.6
Number of radiotherapy sessions	31.44 ± 1.01	31.24 ± 1.01	31.30 ± 0.88	30.22 ± 1.02
Dose per fraction (Gy)	2.2 ± 0.08	2.2 ± 0.08	2.3 ± 0.08	2.2 ± 0.08

**Table 2:** Performance metrics for automatic segmentation of regions of interest. Abbreviations: CET1, contrast-enhanced T1-weighted imaging; T1WI, T1-weighted imaging; T2WI, T2-weighted imaging.

	T1WI		T2WI		CET1	
	Dice Score	IoU	Dice Score	IoU	Dice Score	IoU
Training set	0.709	0.809	0.679	0.791	0.654	0.709
Internal test set	0.644	0.786	0.647	0.824	0.701	0.644
External test set	0.737	0.800	0.666	0.841	0.726	0.737

IoU: Intersection over union.

**Table 3:** Selected radiomics features and their weights.

Imaging Modality	Feature	Weight
T1WI	log-sigma-2-0-mm-3D_firstorder_Skewness	0.0087
	log-sigma-4-0-mm-3D_firstorder_Skewness	0.0070
	log-sigma-4-0-mm-3D_glcm_MCC	−0.0243
	original_shape2D_MajorAxisLength	0.0972
	original_firstorder_InterquartileRange	−0.0198
	original_firstorder_Median	0.0394
	original_firstorder_Range	−0.0352
T2WI	log-sigma-3-0-mm-3D_firstorder_Skewness	0.0040
	log-sigma-4-0-mm-3D_glcm_MCC	−0.0411
	log-sigma-4-0-mm-3D_glrlm_ShortRunEmphasis	0.0005
	log-sigma-4-0-mm-3D_glszm_SmallAreaEmphasis	0.0025
	original_shape2D_MajorAxisLength	0.0729
	original_shape2D_PixelSurface	−0.0321
	original_firstorder_10Percentile	−0.0001
	original_firstorder_Maximum	−0.0049
	original_firstorder_Median	0.0356
	original_firstorder_Range	−0.0484
CET1	original_gldm_GrayLevelNonUniformity	−0.0093
	log-sigma-4-0-mm-3D_glrlm_ShortRunHighGrayLevelEmphasis	0.0251
	log-sigma-4-0-mm-3D_glszm_SmallAreaHighGrayLevelEmphasis	0.0000
	lbp-2D_glrlm_RunVariance	−0.0107
	original_shape2D_MajorAxisLength	0.0585
	original_shape2D_MaximumDiameter	0.0365
	original_shape2D_PixelSurface	−0.0263
	original_firstorder_Maximum	−0.0124
	original_firstorder_Median	0.0399
	original_firstorder_Range	−0.0623
Multi-modal	log-sigma-4-0-mm-3D_firstorder_Skewness (T2WI)	0.0153
	original_shape2D_MajorAxisLength (T2WI)	0.0730
	original_shape2D_PixelSurface (T1WI)	−0.0086
	original_firstorder_InterquartileRange (T1WI)	−0.0029
	original_firstorder_Median (CET1)	0.0292
	original_firstorder_Range (T1WI)	−0.0390

**Table 4:** Evaluation metrics of different imaging modalities across various datasets.

Modality	Dataset	Accuracy	Sensitivity	Specificity	PPV	NPV	F1-Score	AUC
T1WI	Training set	0.762	0.798	0.755	0.361	0.956	0.497	0.773
	Internal test set	0.750	0.741	0.752	0.339	0.944	0.465	0.776
	External test set	0.776	0.829	0.757	0.557	0.923	0.667	0.781
T2WI	Training set	0.760	0.780	0.757	0.357	0.952	0.490	0.752
	Internal test set	0.750	0.704	0.758	0.333	0.937	0.452	0.660
	External test set	0.763	0.780	0.757	0.542	0.903	0.640	0.754
CET1	Training set	0.856	0.881	0.852	0.508	0.976	0.644	0.853
	Internal test set	0.842	0.778	0.854	0.477	0.957	0.592	0.867
	External test set	0.776	0.756	0.784	0.564	0.897	0.646	0.775
Multimodal	Training set	0.955	0.817	0.979	0.873	0.969	0.844	0.905
	Internal test set	0.957	0.815	0.981	0.880	0.969	0.846	0.913
	External test set	0.908	0.805	0.946	0.846	0.929	0.825	0.910

PPV: Positive Predictive Value; NPV: Negative Predictive Value.

**Table 5:** *p*-values for performance differences in various models on different datasets.

Dataset	Statistical Analysis Methods	T1WI vs. T2WI	T1WI vs. CET1	T1WI vs. Multimodal	T2WI vs. CET1	T2WI vs. Multimodal	CET1 vs. Multimodal
Training set	DeLong test	0.371	0.012	<0.001	0.102	<0.001	0.018
	McNemar’s test	0.586	<0.001	<0.001	0.001	<0.001	<0.001
Internal test set	DeLong test	0.149	0.166	<0.001	0.818	0.004	0.012
	McNemar’s test	0.808	0.017	<0.001	0.032	<0.001	<0.001
External test set	DeLong test	0.796	0.833	0.141	0.957	0.066	0.112
	McNemar’s test	0.782	0.662	0.014	0.893	0.008	0.005

## Data Availability

The data presented in this study are available on request from the corresponding author due to privacy and ethical restrictions.

## Supplementary Materials

The following supporting information can be downloaded at: https://www.mdpi.com/article/10.3390/cancers18081265/s1, Figure S1: Probability distribution of T1WI-based model predictions across different datasets (training, internal test, and external test); Figure S2: Probability distribution of T2WI-based model predictions across different datasets (training, internal test, and external test); Figure S3: Probability distribution of CET1-based model predictions across different datasets (training, internal test, and external test); Figure S4: Probability distribution of multimodal-based model predictions across different datasets (training, internal test, and external test); Figure S5: Prediction confusion matrix for the T1WI training set, internal test set, and external test set; Figure S6: Prediction confusion matrix for the T2WI training set, internal test set, and external test set; Figure S7: Prediction confusion matrix for CET1 training set, internal test set, and external test set; Figure S8: Prediction confusion matrix of the fusion model training set, internal test set, and external test. Figure S9: Bar chart depicting the AUC drift with and without Pearson Correlation Filtering across identical data partitions. Error bars denote the 95% Confidence Intervals. Figure S10: illustrates the model ablation study on similar features. Evaluating identical data partitions revealed that the isolated removal of Interquartile Range and Range (retaining Median) degraded model performance, yielding AUCs of 0.892, 0.890, and 0.882 in the training, internal test, and external validation cohorts, respectively (compared to baseline values of 0.905, 0.913, and 0.910). CIs and DeLong verification ensure systemic confidence in multi-scale independence. Figure S11: Comparative analysis outlining optimal shrinkage selection accuracy across the Training, Internal Test, and External Validation cohorts. Statistical 95% CIs distinguish systematic advantages inherent to LASSO deployment models. Figure S12: Multi-cohort evaluation (Training, Internal, and External) and stable AUC performance corresponding to ComBat empirical Bayes harmonization targeting 1.5 T and 3.0 T magnetic resonance scanners differences. Confidence bars designate 95% CIs demonstrating highly consistent performance. Figure S13: Bar chart showcasing the comparable predictive performance when transitioning from expert-reviewed regions (Manual ROIs) to fully autonomous deep learning masks (Automatic Segmentations). Marginal error bars display 95% Confidence Intervals. Figure S14: Comparative performance analysis illustrating the reduction in external generalizability when omitting the standardized isotropic spatial resampling preprocessing step. Error bars reflect 95% Confidence Intervals. Table S1: Feature counts retained at each step of the radiomics preprocessing pipeline. Table S2: Variance inflation factor values for the final selected multimodal features.
