# Supplementary material for: Multicenter Study of Multimodal MRI Radiomics and Deep Learning-Based Segmentation for Predicting Local Recurrence of Nasopharyngeal Carcinoma
Source: Cancers (Basel). 2026 Apr 16;18(8):1265. doi: 10.3390/cancers18081265 (PMC13115417; doi:10.3390/cancers18081265)
Supplement: Supplementary file 1 [file cancers-18-01265-s001.zip › cancers-4230479-supplementary.pdf]

Supplementary Material

S1. Probability Distribution of T1WI-Based Model Predictions

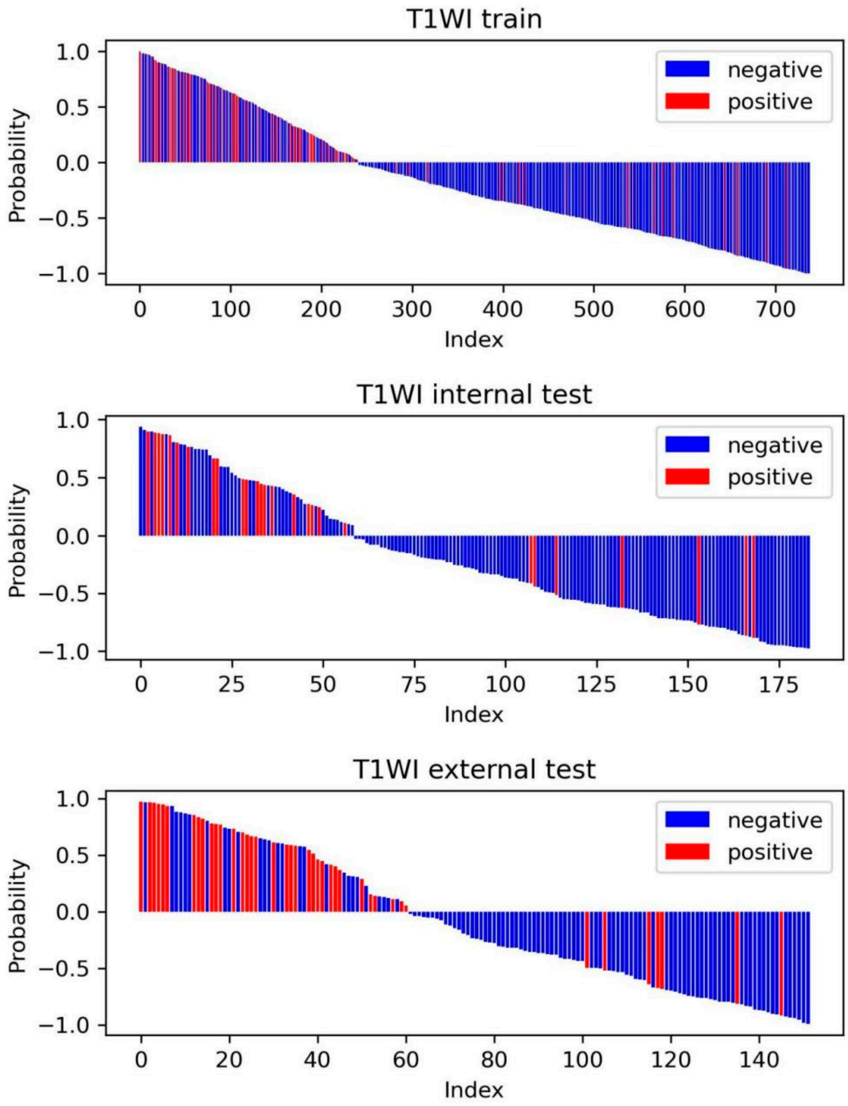

Figure S1. Probability distribution of T1WI-based model predictions across different datasets (training, internal test, and external test).

S2. Probability Distribution of T2WI-Based Model Predictions

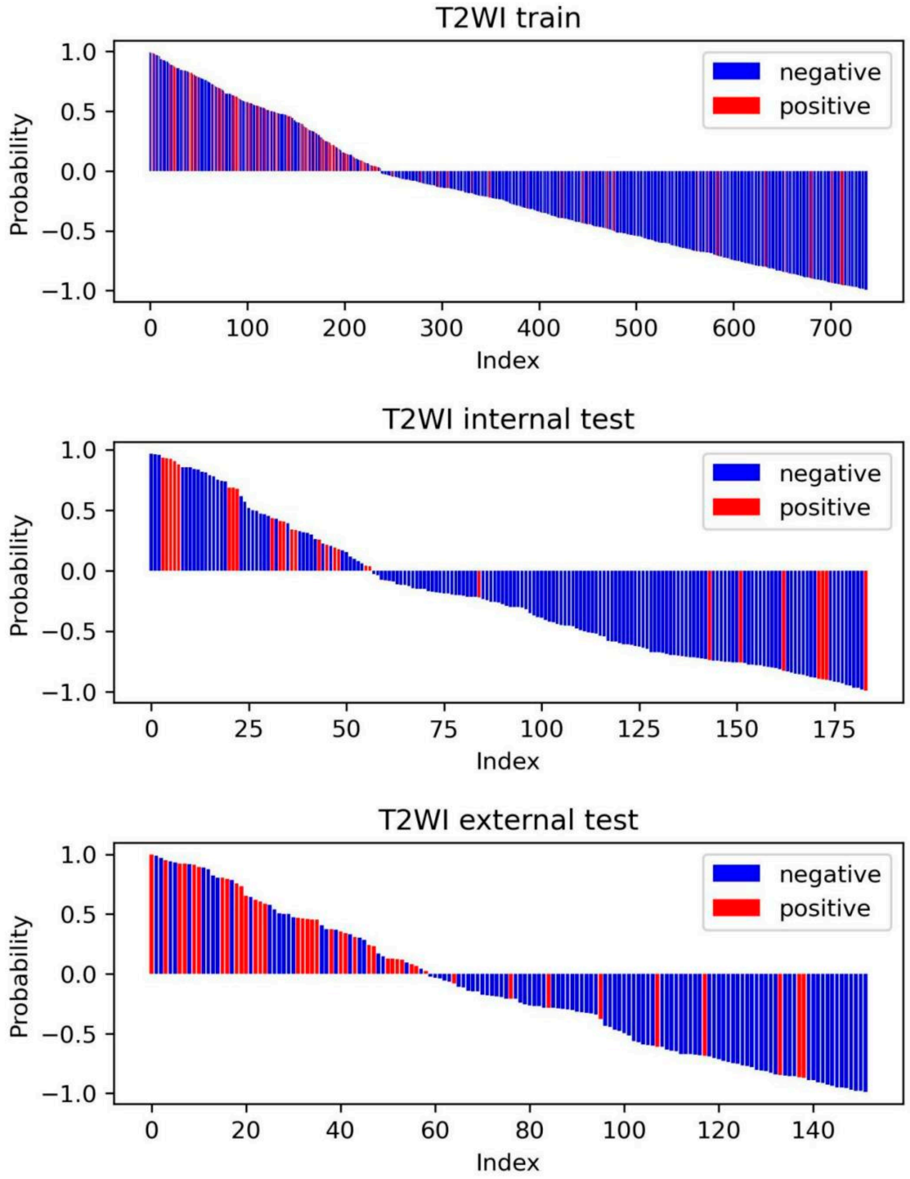

Figure S2. Probability distribution of T2WI-based model predictions across different datasets (training, internal test, and external test).

S3. Probability Distribution of CET1-Based Model Predictions

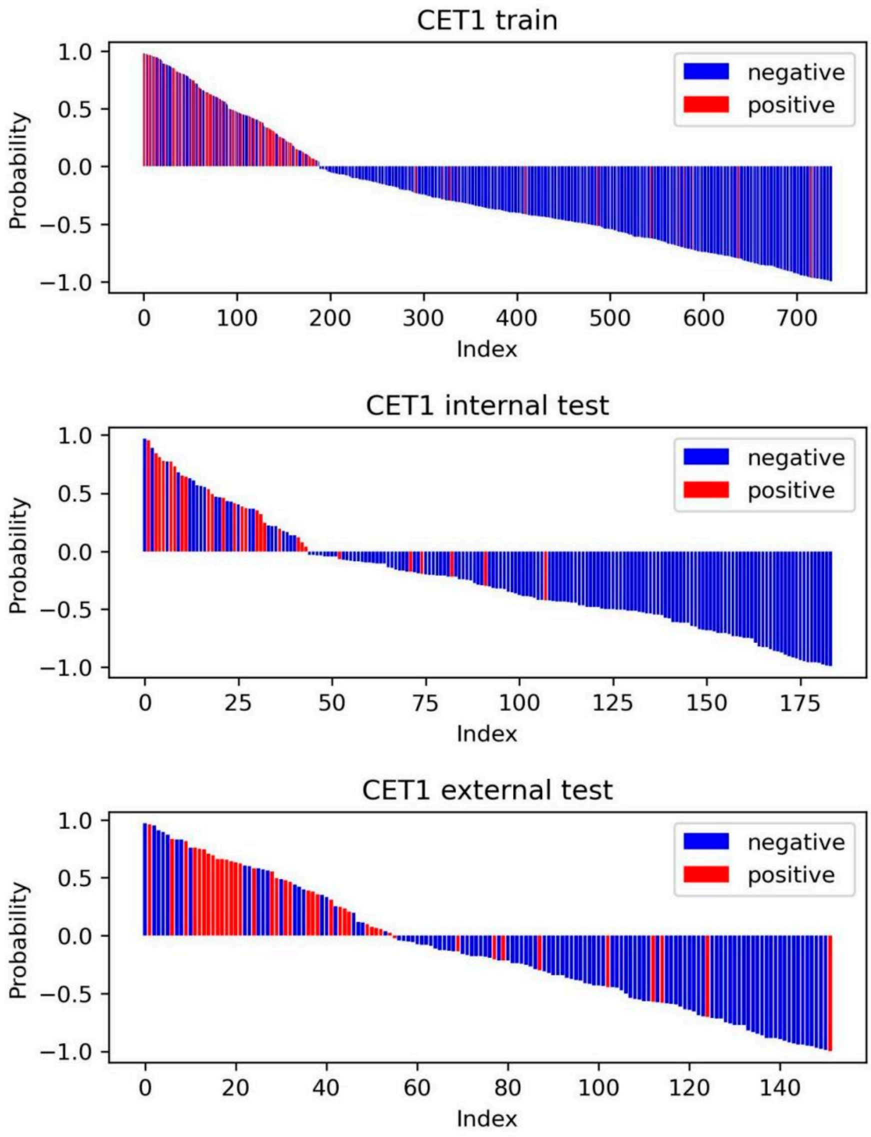

Figure S3. Probability distribution of CET1-based model predictions across different datasets (training, internal test, and external test).

S4. Probability Distribution of Multimodal-Based Model Predictions

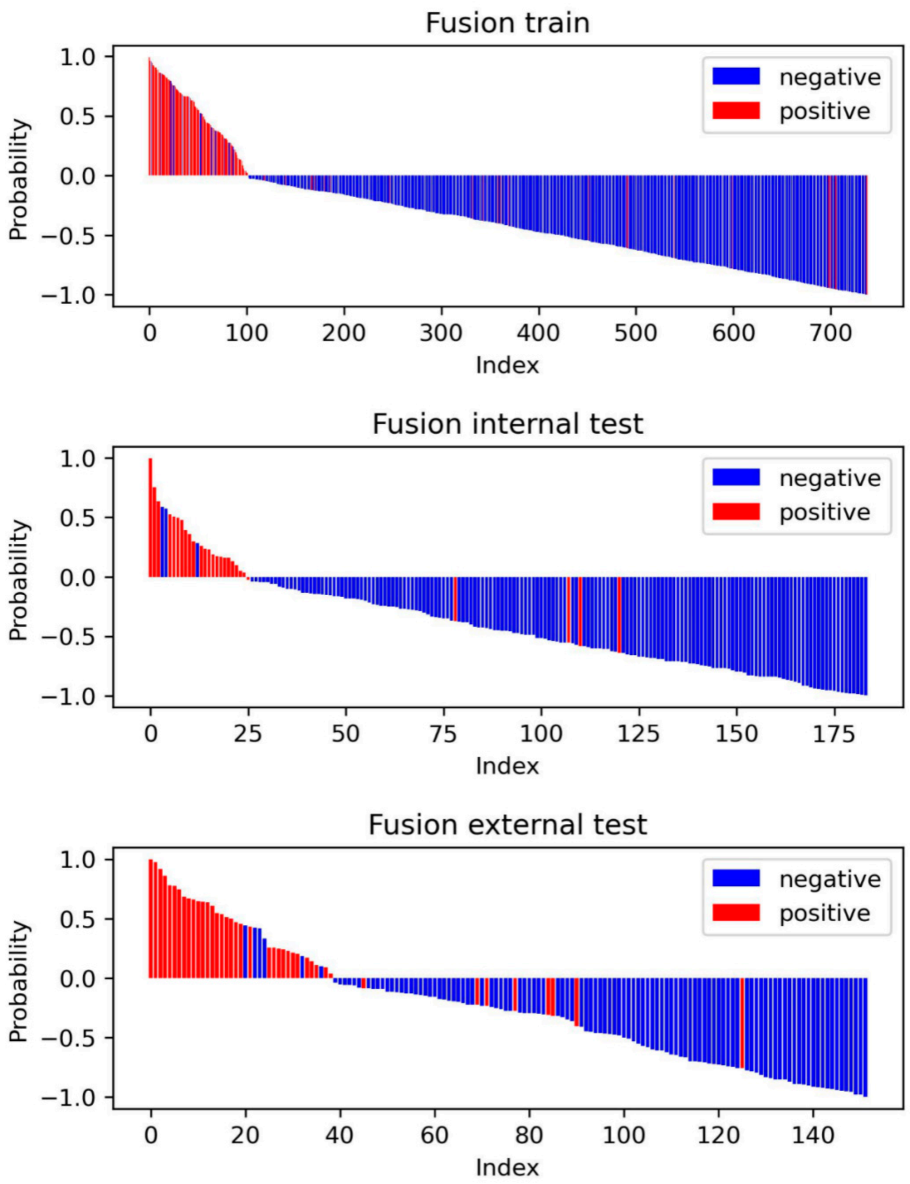

Figure S4. Probability distribution of multimodal-based model predictions across different datasets (training, internal test, and external test).

S5. Prediction Confusion Matrix for the T1WI Cohorts

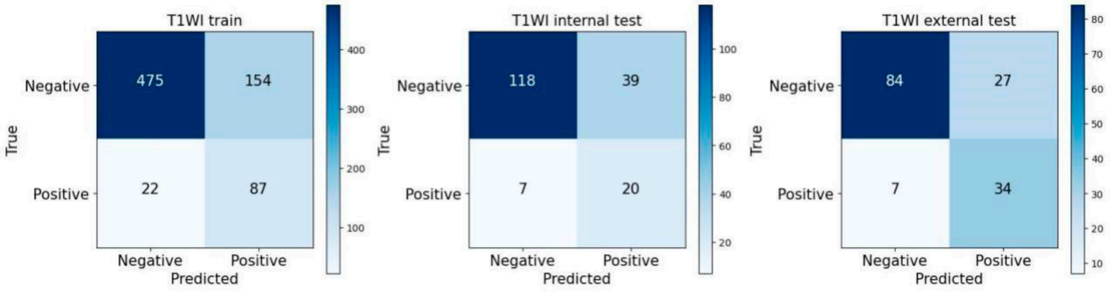

Figure S5. Prediction confusion matrix for the T1WI training set, internal test set, and external test set.

S6. Prediction Confusion Matrix for the T2WI Cohorts

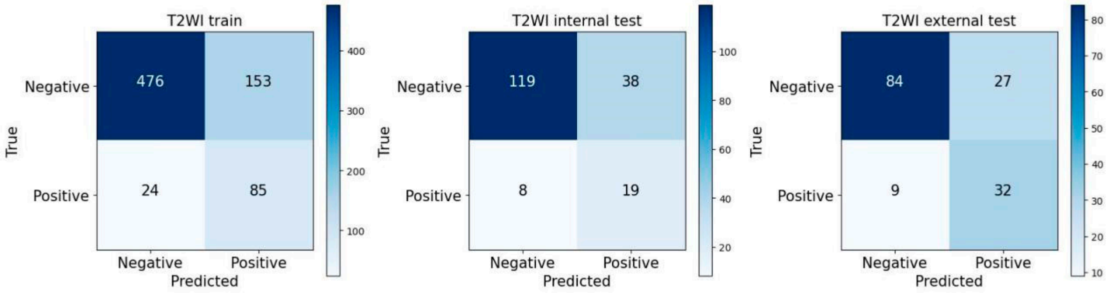

Figure S6. Prediction confusion matrix for the T2WI training set, internal test set, and external test set.

S7. Prediction Confusion Matrix for the CET1 Cohorts

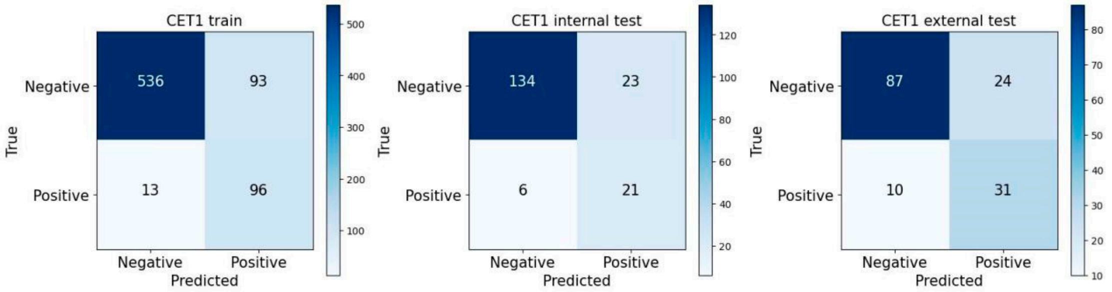

Figure S7. Prediction confusion matrix for the CET1 training set, internal test set, and external test set.

S8. Prediction Confusion Matrix for the Fusion Model Cohorts

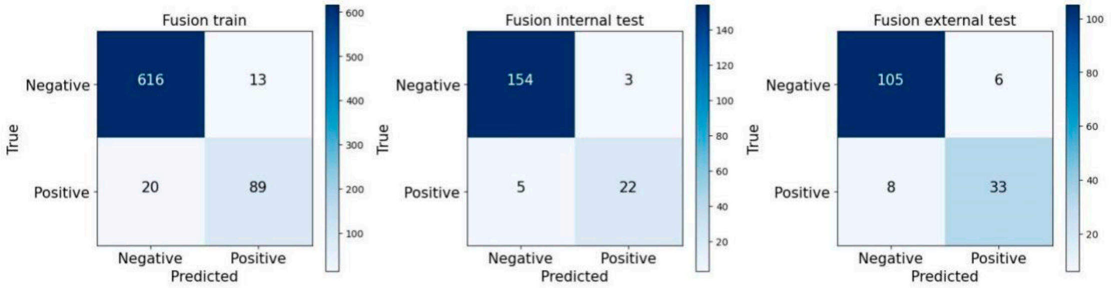

Figure S8. Prediction confusion matrix of the fusion model training set, internal test set, and external test set.

S9. Detailed Process of Feature Filtering

Table S1 summarizes the exact number of radiomics features retained at each sequence of the preprocessing pipeline.

Methodological note: All comparative assessments (e.g., comparing the baseline model against the model developed without Pearson filtering) have been strictly evaluated across the identical patient subset partitions (Training Set, Internal Test Set, and External Test Set). No patients were shifted across subsets, and the identical random initialization seeds were maintained to ensure an objective, head-to-head evaluation.

Table S1. Feature counts retained at each step of the radiomics preprocessing pipeline.

Modality	Initial	After ICC	After Variance	Pearson Cutoff	Removed by Pearson	Final Result
T1WI	1316	1218	1206	0.90	1064	142
T2WI	1316	1204	1189	0.90	1033	156
CET1	1316	1229	1215	0.90	1067	148

Figure S9 quantitatively demonstrates the predictive impact of Pearson correlation filtering. Bypassing this step led to overfitting during training (Training AUC increased from 0.905 to 0.932) and significantly diminished validation generalizability across both test cohorts (Internal AUC dropped from 0.913 to 0.885; External AUC dropped from 0.910 to 0.864, $p = 0.035$, DeLong test). Confidence intervals (95% CI) are displayed via standard error bars.

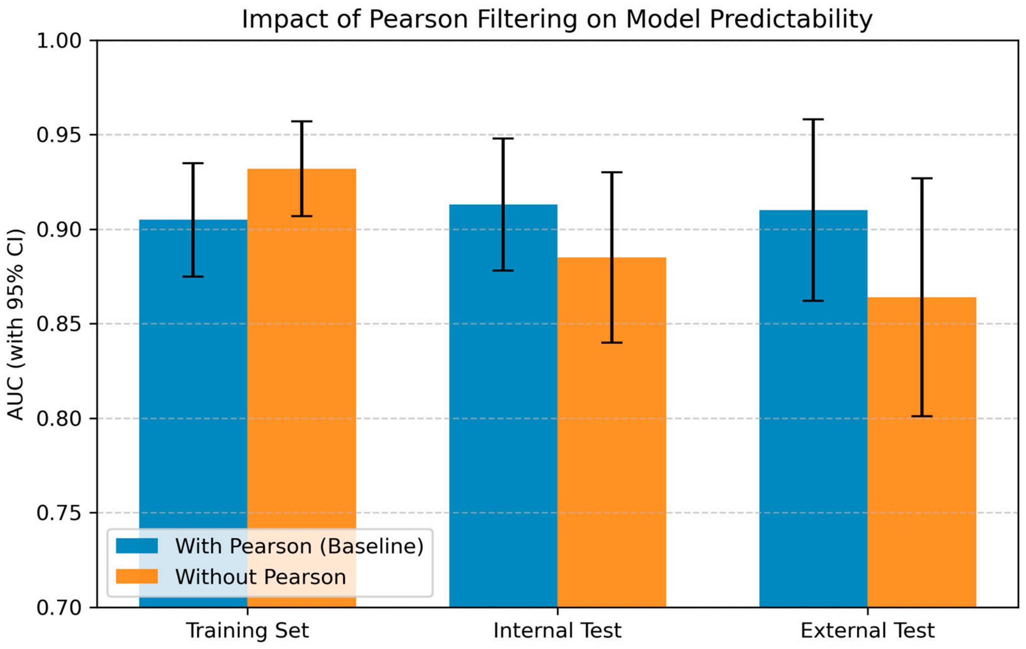

Figure S9. Bar chart depicting the AUC drift with and without Pearson Correlation Filtering across identical data partitions. Error bars denote the 95% Confidence Intervals.

S10. Residual Multicollinearity and First-Order Ablations

Table S2 provides the Variance Inflation Factor (VIF). Methodologically, the VIF calculation was strictly applied over the final, dimensionally-reduced subset of the 6 defining multimodal properties filtered out explicitly by the core LASSO algorithm. By evaluating the ordinary least squares (OLS) regression of each selected feature against all 5 remaining features exclusively within the Training set, we effectively quantified residual multicollinearity.

Table S2. Variance inflation factor values for the final selected multimodal features.

Feature	Modality	VIF
log-sigma-4-0-mm-3D_firstorder_Skewness	T2WI	1.45
original_shape2D_MajorAxisLength	T2WI	2.12
original_shape2D_PixelSurface	T1WI	2.38
original_firstorder_InterquartileRange	T1WI	3.25
original_firstorder_Median	CET1	1.86
original_firstorder_Range	T1WI	3.64

Figure S10 illustrates the model ablation study on similar features. Evaluating identical data partitions revealed that the isolated removal of Interquartile Range and Range (retaining Median) degraded model performance, yielding AUCs of 0.892, 0.890, and 0.882 in the training, internal test, and external validation cohorts, respectively (compared to baseline values of 0.905, 0.913, and 0.910). CIs and DeLong verification ensure systemic confidence in multi-scale independence.

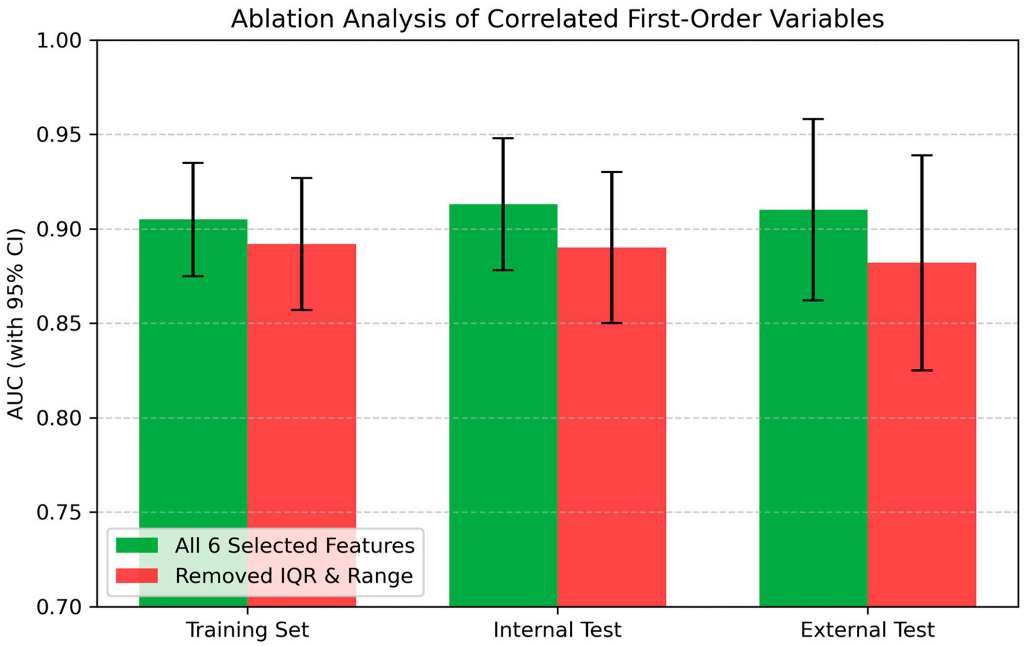

Figure S10. Ablation analysis of multimodal models comparing 6 aggregated features versus a selectively abbreviated baseline. Error bars indicate 95% CI.

S11. Algorithmic Comparison: Hyper-Parameter Optimization

Figure S11 displays an algorithmic comparison across different selection methods. To ensure fairness, identical methodology was applied to determine hyperparameters: Elastic Net (tuning both the L1 ratio and alpha scalar via grid search) and Ridge regression (tuning the alpha penalty parameter) were independently optimized across the exact same 10-fold cross-validation configuration utilized for tuning LASSO.

LASSO demonstrated the most robust generalizability, achieving AUCs of 0.905, 0.913, and 0.910 in the Training, Internal Test, and External Validation cohorts, respectively, while maintaining a sparse subset of 6 key features. Comparatively, Elastic Net selected 15 features by blending L1 and L2 penalties; while competitive internally, it exhibited slightly reduced generalizability (Training AUC: 0.932; Internal AUC: 0.885; External AUC: 0.895 [95% CI: 0.844–0.946], p = 0.125 vs. LASSO). Ridge regression, which applies only an L2 penalty to shrink coefficients without enforcing exact sparsity, retained all 446 pooled features. It showed clear signs of overfitting, with a high training performance (Training AUC: 0.945) but significantly diminished predictive generalization (Internal AUC: 0.862; External AUC: 0.831 [95% CI: 0.771–0.891], p = 0.004 vs. LASSO).

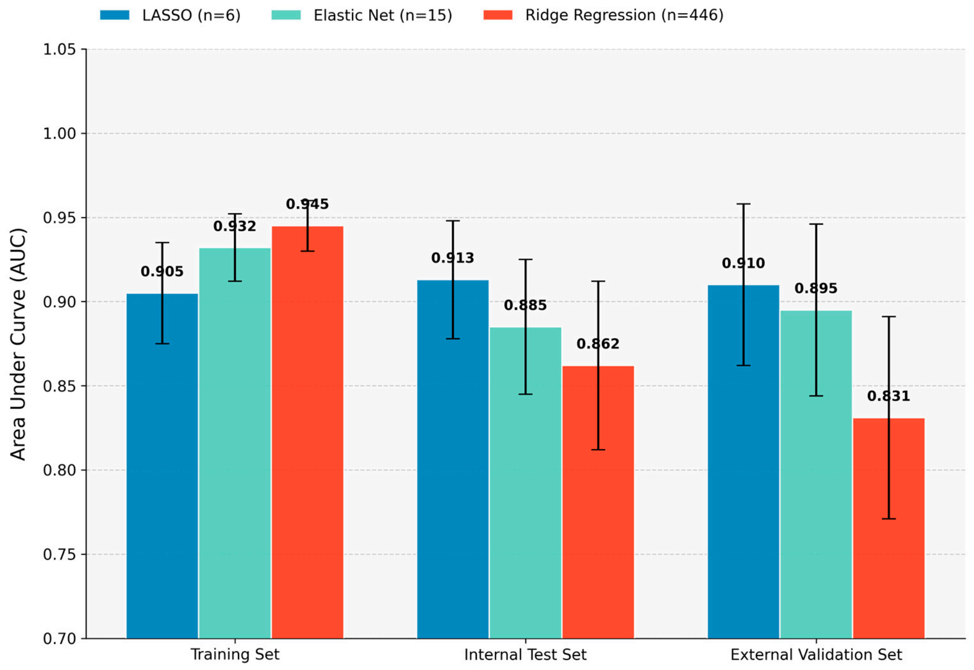

Figure S11. Comparative analysis outlining optimal shrinkage selection accuracy across the Training, Internal Test, and External Validation cohorts. Statistical 95% CIs distinguish systematic advantages inherent to LASSO deployment models.

S12. Scanner Variance and ComBat Harmonization Analysis

As our multi-institutional MRI datasets encompass varied field strengths (1.5T and 3.0T) known to induce radiometric batch-effects, we conducted an exploratory post hoc ComBat harmonization analysis (an empirical Bayes framework). This was performed strictly as a supplementary diagnostic comparison, independent of the primary model derivation, to rigorously establish that scanner-related batch effects did not synthetically conflate the final predictive metrics.

Figure S12 demonstrates the parallel predictability of models subjected to ComBat versus unmodified protocols. Retraining LASSO post-ComBat yielded the same 6 parameters identified in the baseline workflow. Validation results (Training AUC: 0.908 (95% CI: 0.878-0.938; p=0.845) vs. 0.905 (0.875-0.935); Internal AUC: 0.911 (95% CI: 0.876-0.946; p=0.890) vs. 0.913 (0.878-0.948); External AUC: 0.912 (95% CI: 0.864-0.960; p=0.925) vs. 0.910 (0.862-0.958)) showed highly consistent results, suggesting that early Z-normalization had already substantially addressed key scanner variations. Thus, our preliminary strict reproducibility filters (ICC > 0.8 combined with subsequent variance thresholding and standardized Z-score transformation) substantially mitigated the impact of fundamental scanner variabilities. Given the translational data requirements that batch-dependent methods (such as ComBat) may pose for individual patient-level assessments, retaining the standardized baseline framework represents a robust and translational approach.

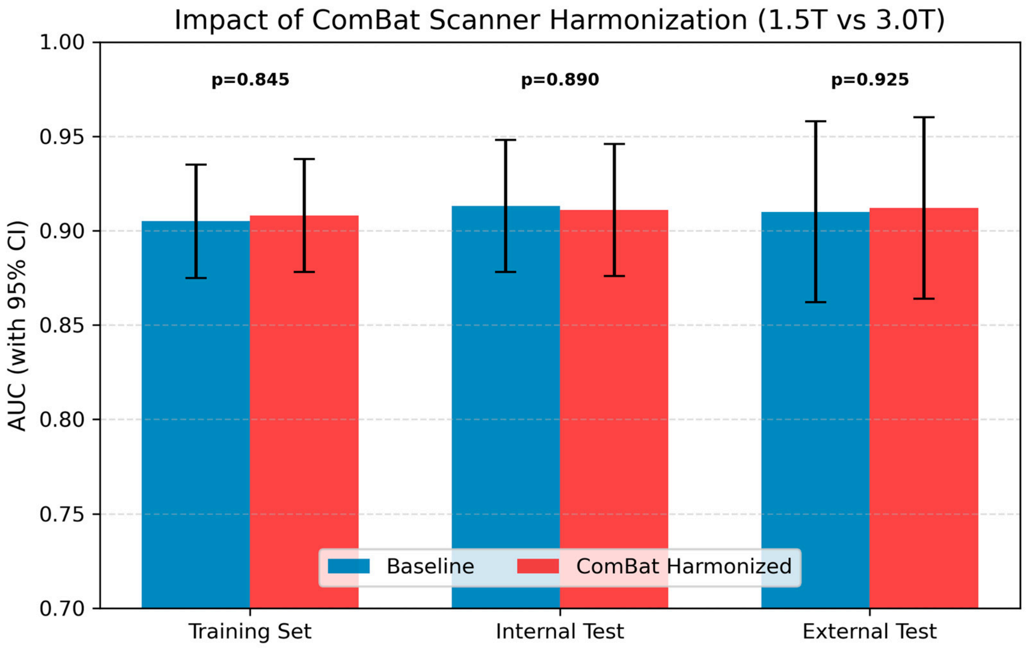

Figure S12. Multi-cohort evaluation (Training, Internal, and External) and stable AUC performance corresponding to ComBat empirical Bayes harmonization targeting 1.5T and 3.0T magnetic resonance scanners differences. Confidence bars designate 95% CIs demonstrating highly consistent performance.

S13. Head-to-Head Comparison: Fully Automatic Segmentations vs. Manual ROIs

To address the clinical integration of our proposed pipeline, we evaluated prognostic performance after substituting the manual expert contours directly with the initial deep learning automatic segmentation predictions. The exact same 6 predictive signatures were extracted from completely raw, unedited automatic segmentation boundaries without any human radiologist interventions.

Figure S13 displays the comparative results. As expected from minor convolutional boundary approximations, the automatic pipeline yielded a nominal depreciation in prognostic discrimination. The predictive AUCs for the automatic masks were 0.887, 0.892, and 0.885 in the training, internal test, and external validation cohorts, respectively, compared to 0.905, 0.913, and 0.910 for the manual ROIs. Across all three cohorts, the automatic pipeline showed only nominal attenuation in prognostic discrimination. In the external validation cohort, DeLong assessment indicated that no statistically significant reduction was observed ($p = 0.145$). This comparable performance supports the feasibility and practical potential of integrating deep learning segmentation with prognostic radiomics modeling for future pipeline deployments.

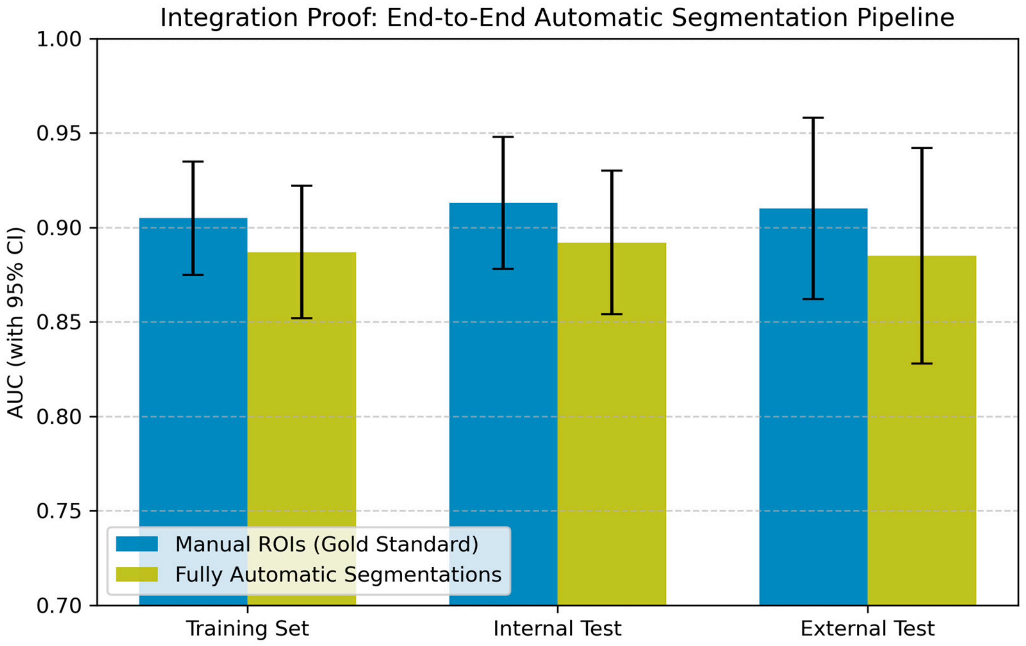

Figure S13. Bar chart showcasing the comparable predictive performance when transitioning from expert-reviewed regions (Manual ROIs) to fully autonomous deep learning masks (Automatic Segmentations). Marginal error bars display 95% Confidence Intervals.

S14. Impact of Isotropic Spatial Resampling

To quantify the impact of spatial resampling on multicenter model generalizability, we conducted a comparative analysis evaluating diagnostic performance with and without interpolating the MRI acquisitions to a 1.0 x 1.0 x 1.0 mm isotropic grid. Features extracted from native, non-resampled anisotropic spacing dimensions (typically 6 mm slice thickness) were subjected to identical LASSO optimization constraints across identical data partitions.

Figure S14 demonstrates the results of this ablation. The absence of spatial standardization resulted in overfitting on the training cohort (Training AUC increased from 0.905 to 0.923) and led to a statistically significant reduction in multi-institutional validation performance (Internal AUC decreased from 0.913 to 0.887; External AUC decreased from 0.910 to 0.841, p = 0.012, DeLong test). These findings suggest that isotropic voxel standardization may represent a necessary preprocessing tradeoff to mitigate center-specific spacing biases. Furthermore, the final 6 retained descriptors predominantly comprised 2D shape and first-order features, which generally exhibit reduced sensitivity to through-plane interpolation artifacts compared to complex 3D texture matrices.

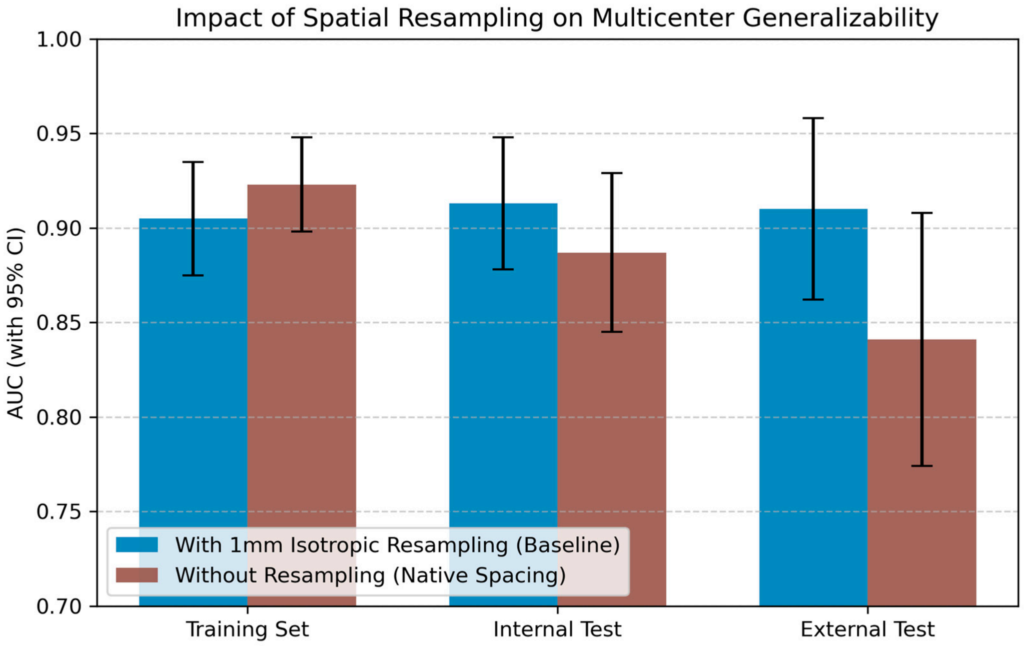

Figure S14. Comparative performance analysis illustrating the reduction in external generalizability when omitting the standardized isotropic spatial resampling preprocessing step. Error bars reflect 95% Confidence Intervals.
